# Enhancer Trapping and Annotation in Zebrafish Mediated with *Sleeping Beauty*, *piggyBac* and *Tol2* Transposons

**DOI:** 10.3390/genes9120630

**Published:** 2018-12-13

**Authors:** Dan Shen, Songlei Xue, Shuheng Chan, Yatong Sang, Saisai Wang, Yali Wang, Cai Chen, Bo Gao, Ferenc Mueller, Chengyi Song

**Affiliations:** 1 Institute of Mobilome and Genome, College of Animal Science & Technology, Yangzhou University, Yangzhou 225009, China; shendan2009@hotmail.com (D.S.); xuesl2013@hotmail.com (S.X.); 15380345581@163.com (S.C.); sangyatong@hotmail.com (Y.S.); wang850980245@hotmail.com (S.W.); lilies2016@outlook.com (Y.W.); chencai9596@hotmail.com (C.C.); bgao@yzu.edu.cn (B.G.); 2 Institute of Cancer and Genomic Sciences, College of Medical and Dental Sciences, University of Birmingham, Edgbaston, Birmingham B15 2TT, UK; F.Mueller@bham.ac.uk

**Keywords:** enhancer trapping, enhancer, *Sleeping Beauty*, *piggyBac*, *Tol2*, zebrafish

## Abstract

Although transposon-mediated enhancer trapping (ET) is successfully applied in diverse models, the efficiency of various transposon systems varies significantly, and little information is available regarding efficiency of enhancer trapping by various transposons in zebrafish. Most potential enhancers (Ens) still lack evidence of actual En activity. Here, we compared the differences in ET efficiency between *sleeping beauty* (*SB*), *piggyBac* (*PB*) and *Tol2* transposons. *Tol2* represented the highest germline transfer efficiencies at 55.56% (N_F0_ = 165), followed by *SB* (38.36%, N_F0_ = 151) and *PB* (32.65%, N_F0_ = 149). ET lines generated by the *Tol2* transposon tended to produce offspring with a single expression pattern per line, while *PB* and *SB* tended to generate embryos with multiple expression patterns. In our tests, 10 putative Ens (En1–10) were identified by splinkerette PCR and comparative genomic analysis. Combining the *GFP* expression profiles and mRNA expression patterns revealed that En1 and En2 may be involved in regulation of the expression of *dlx1a* and *dlx2a*, while En6 may be involved in regulation of the expression of line TK4 transgene and *rps26*, and En7 may be involved in the regulation of the expression of *wnt1* and *wnt10b*. Most identified Ens were found to be transcribed in zebrafish embryos, and their regulatory function may involve eRNAs.

## 1. Introduction

The term ‘enhancer’ (En) was first introduced to describe the effects of SV40 DNA on the ectopic expression of the gene for a cloned rabbit β-globin [1]. Ens can mediate the on/off switch patterns of gene expression in specific cell types at particular stages during animal embryogenesis and development [2]. Ens can act at a long distance away from their target gene and independent of their orientation [3]; some Ens have also been shown to be involved in genetic disease in humans [4,5]. These functional properties make Ens an important part of the genomic regulatory architecture.

The methods of identification of novel Ens are continuously improving, yet the typical En trapping (ET) technology is based on a randomly inserted transposon-mediated vector, which harbors a trapping box containing a mini-promoter, reporter open reading frame (ORF) and polyA, and the trapping box is flanked by inverse terminal repeats (ITRs) of transposon [6,7,8,9,10]. As the development of retrovirus- and transposon-based technologies for gene transfer progresses, the efficiency of integrating reporters into the genome has been improved significantly [11]. Transposons represent the most effective insertion technology in vertebrates to date, and have been developed as gene delivery vectors for gene therapy, insertional mutagenesis and other experimental approaches. Numerous studies of transposon-mediated En detection have been reported for medaka [12], zebrafish [13,14,15], mice [16], and insects [17]. A multifunctional mutagenesis system in zebrafish using the maize Ds transposon has been described, in which ET efficiency as measured by reporter green fluorescent protein (*GFP*) expression frequency was 23% [18]. A *piggyBac* (*PB*) transposon-based ET system developed for a human malaria vector resulted in 314 progeny arising with ET [19]. Subsequently, *PB*-mediated ET was efficiently used for discovering and manipulating neuronal cell types [20]. Large-scale analysis of the regulatory architecture of the mouse genome with a *SB* transposon-associated sensor generated several hundred mice and embryos, each with a regulatory sensor inserted at a random genomic position [21]. The *SB* transposon system has also been tested for ET in zebrafish, and nine transgenic zebrafish lines with distinct tissue-specific *GFP* expression patterns were obtained, but only one En (*mkp3*) was finally identified [13]. *Tol2* transposons have been extensively applied for ET in zebrafish [22,23]. Zebrafish ET transgenics performed with the *Tol2* transposon technique could be instrumental for studies of the development of the circumventricular organs in zebrafish and for understanding the molecular mechanisms of diseases such as hydrocephalus in humans [24]. Twelve zebrafish lines with a single copy and tissue- or cell-specific *GFP* expression were obtained using a *Tol2* transposable system containing a minimal mouse metallothionein gene promoter and *GFP*, and one potential En element was identified [11]. *Tol2* transposon-mediated gene trap and ET screens found a subpopulation of neurons in the zebrafish dorsal telencephalon (Dm) essential for fear conditioning, which were called 120A-Dm neurons. Inhibition of the 120A-Dm neurons caused reduced performance in Pavlovian fear conditioning [25]. The *Tol2* insertions were also mobilized efficiently in the germline, and a *Tol2* gene trap construct mobilized an insertion into *nup214* [26]. As an alternative to traditional ET methods, a binary Gal4-UAS system has been used successfully for ET construction and monitoring cell- or tissue-specific expression of reporter genes in *Drosophila* and zebrafish [16,27,28,29].

Preference of target sites of transposons may play roles in influencing ET efficiency. Target site preferences of different transposons range from essentially random to selective with respect to the actual DNA sequences, at which integrations occur, but are invariably non-random on the genome-wide scale [30]. The *SB* element does not appear to exhibit a pronounced preference for targeted sequence [31], and it tends to generate predominantly single-copy insertion [30]. However, both *PB* and *Tol2* have been found to be biased towards transcriptional units, CpG islands and transcription start sites (TSSs), open chromatin in general [32,33,34], and as a result, target site preferences may have a significant influence on ET efficiency.

Despite the success of various transposon-mediated ETs in a number of species, including ET mediated with *SB*, *Tol2* and *Ds* applied in zebrafish, there is a lack of direct comparison of ET efficiency between the various transposons in zebrafish. Moreover, few studies have tested the actual enhancer activity of trapped potential Ens. Here, we compared the differences in ET efficiency, including transient *GFP* expression frequency, germline transmission efficiency and the diversity of captured patterns, among *PB*, *SB*, and *Tol2* transposons. We have annotated the Ens trapped by stable transgenic lines, and detected the actual activity of each trapped En in reporter constructs in vivo. The transposon mediated ET methods and results described here represent a systematic approach to discover and evaluate *cis*-regulatory activity and aim to enhance our understanding of transcriptional regulation during vertebrate embryonic development.

## 2. Materials and Methods

### 2.1. Vector Construction, Injection and Transgenic Screening

**Figure 1:**
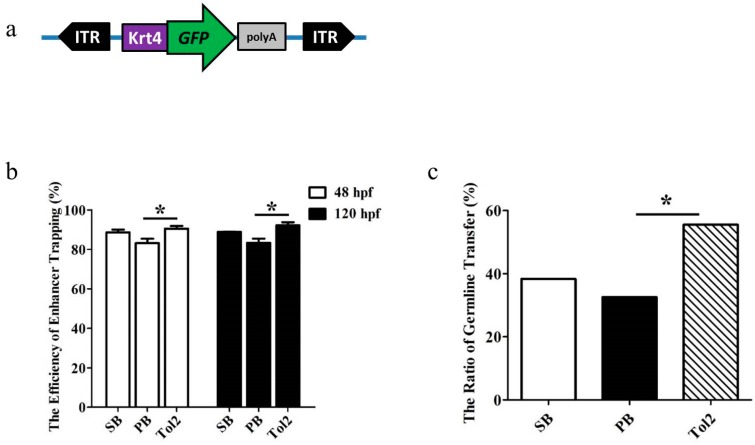
*Green fluorescent protein (GFP)* expression efficiency and germline transfer efficiency of enhancer trapping (ET) based on *sleeping beauty* (*SB*), *piggyBa* (*PB*) and *Tol2* transposons in zebrafish. (**a**) Schematic of the transposon-based ET cassette (not to scale). The cassette includes two inverted repeat sequences (*SB*, *PB*, or *Tol2* inverse terminal repeats (ITRs)), and a minimal Krt4 promoter, *GFP*, and β-globin polyA signal; (**b**) *GFP* expression efficiency was measured by *GFP* screen at 48 and 120 h postfertilization (hpf) after co-injection of a fixed amount of transposon plasmid with the corresponding transposase mRNA into zebrafish embryos. The total number of injected embryos was 1378 in *SB*, 1276 in *PB*, 1524 in *Tol2*. Error bars represent SD; (**c**) germline efficiency was measured by crossing the *GFP*-positive founders with wild-type (WT) fish. Significant difference was detected using a χ-squared test; asterisk indicates *p* < 0.05.

To compare the efficiency of ET by *SB*, *PB* and *Tol2* transposons, the *GFP* trapping box containing mini-promoter Krt4 (*keratin4*), *GFP* and β-globin polyA was constructed. The Krt4 minimal promoter was cloned from the zebrafish genome using primers 5′-gcACTAGTAAGCTTgtgtgtgtgtgagagcagtc-3′ and 5′-atGAATTCaggtacgagagtgctctctg-3′, and the CAG promoter of pCAG-*GFP* vector (Addgene: 11150) was replaced with *Spe*I/*Eco*RI sites. Then, the *GFP* trapping box was subcloned into the *SB*, *PB* and *Tol2* transposon structural frames and named p*SB*-Krt4-*GFP*, p*PB*-Krt4-*GFP* and p*Tol2*-Krt4-*GFP*, respectively (Figure 1a). The *SB100X* transposase plasmid was a gift from Dr. Zoltan Ivics (Paul Ehrlich Institute, Germany); the *PB* transposase plasmid was a gift from Dr. Allan Bradley (Wellcome Trust Sanger Institute, UK); the *Tol2* transposase plasmid was received from Vladim Korzh (National University of Singapore, Singapore); the *SB100X* and *PB* and *Tol2* ORFs were subcloned into the pTNT^TM^ vector, while pTNT^TM^ plasmid was ordered from (AL5610; Promega, Fitchburg, WI, USA). We named them pTNT-*SB100X,* pTNT-*PB* and pTNT-*Tol2*. Then, transposase mRNA was synthesized in vitro using a mMESSAGE mMACHINE T7 Kit (AM1344; Ambion, Austin, Texas, USA) from the pTNT-*SB100X*/*PB*/*Tol2* constructs according to the kit instructions.

### 2.2. Injection of Fertilized Eggs

To compare the *GFP* expression frequency of the three transposons, transposon donor plasmids (p*SB*-Krt4-*GFP*, p*PB*-Krt4-*GFP*, and p*Tol2*-Krt4-*GFP*) at 20 ng/µL were mixed with capped transposase mRNA at 50 ng/µL for SB and PB, or at 30 ng/µL for *Tol2* respectively. In each batch of experiments, 10–20 pairs of parental fish were used to naturally produce fertilized eggs and these eggs were evenly allocated to the three transposon groups for microinjection. Then, the mixture was injected into individual zebrafish fertilized in the yolk cell. There were approximately 500 eggs in each group. Three batches of independent experiments were repeated to obtain statistically significant results. After injection, *GFP* expression was screened at 48 and 120 h postfertilization (hpf) by fluorescence microscopy. *GFP*-positive embryos at 120 hpf were raised to adulthood and crossed to (wild-type) WT for *GFP* germline transfer analysis, and the *GFP* expression of F1 embryos was screened at 1, 2, 3, 4, 5, 6 and 7 days postfertilization (dpf) under the Leica (Solms, Germany) M165 FC fluorescent microscope.

Zebrafish were maintained at 28.5 °C in a licensed aquarium facility (ESEN, Beijing, China) according to standard protocols. All treatments and protocols involving zebrafish in this study were strictly done in accordance with the guidelines of the Animal Experiment Ethics Committee of Yangzhou University (approval number:YZUDWSY2018-12).

### 2.3. Amplification of Transposon Insertion Sites

To clarify the genetic backgrounds corresponding to ET lines with specific *GFP* expression patterns, we detected insertion events in these lines. First, *GFP*-positive F0 fish were outcrossed with WT fish to obtain *GFP*-positive offspring (F1 fish) for stable transgenic lines. Then, F1 fish were outcrossed with WT fish to generate positive offspring for splinkerette PCR assay according to the protocol described previously, and each line was outcrossed five times to observe the segregation of expression pattern, and at least three batches of embryos from different outcrossing were used for splinkerette PCR [35]. Genomic DNAs were digested with *Sau*3AI, followed by two rounds of PCR amplification with primers specific for the transposon and splinkerette (Table S1). The first-round PCR was performed using the digested genomic DNA as the template with the primer pairs of SPLINK1/TN-1R under the following conditions: 1 cycle at 94 °C for 5 min; 35 cycles at 94 °C for 30 s, 60 °C for 30 s, 72 °C for 2 min; 1 cycle at 72 °C for 10 min. Then, the first-round PCR product (1 µL) was used as a template for the second-round PCR with primer pairs SPLINK2/TN-2R under the following conditions: 1 cycle at 94 °C for 5 min; 35 cycles at 94 °C for 30 s, 58 °C for 30 s, 72 °C for 1 min 30 s; 1 cycle at 72 °C for 10 min. The products of the second round of PCR amplification were purified and submitted for sequencing using primer T1.

### 2.4. Computational Analysis of Genomic Sequences

To identify the putative Ens, the chromosomal flanking DNA sequences around the insertion sites were mapped on the zebrafish genome sequence (GRCz10) in the Ensembl (http://asia.ensembl.org) by BLASTN (https://blast.ncbi.nlm.nih.gov/Blast.cgi), then genomic sequences spanning the region between the target genes from diverse representative species of vertebrates were downloaded from the Ensembl browser, and multiple alignments of genomic sequences were made using mVISTA (http://genome.lbl.gov/vista/mvista/submit.shtml) to identify the conserved non-coding sequences, which are putative Ens.

### 2.5. Whole-Mount RNA In Situ Hybridization and Transcription Analysis of Identified Ens

To identify the expression profile of the endogenous gene near the insertion site, whole-mount RNA in situ hybridization (WISH) of the nearest gene was performed as previously described [36]. Antisense RNA probes for *dlx1a*, *dlx2a*, *wnt1*, *wnt10b*, *rps26* and *ednraa* were synthesized by using digoxigenin (Roche, Basel, Switzerland) as a label. Images were captured using the M165 FC fluorescent microscope (Leica, Solms, Germany).

To investigate the transcription of the identified Ens, the embryos, developed to 24 and 48 hpf (hours post fertilization), were used for reverse transcription (RT)-PCR and real-time quantitative-PCR (qPCR) (the primers for qPCR are listed in Table S2).

### 2.6. Enhancer Activity Analysis In Vivo

The newly identified Ens were cloned from the zebrafish genome with the primers listed in Table S1 and inserted into an En test vector to retest the expression dynamics. The En test vector contained the gata mini-promoter, *GFP* ORF, and β-globin polyA flanked with two insulators; we named the new vector including Ens as pEn-gata-*GFP*. Then, the plasmid (pEn-gata-*GFP*) at 20 ng/µL containing Ens was mixed with capped *Tol2* transposase mRNA at 30 ng/µL injected into individual zebrafish embryos at the one-cell stage. After injection, embryos were screened at 24 and 48 hpf.

## 3. Results

### 3.1. Comparative Analysis of the Enhancer Trapping Efficiency of *PB*, *SB* and *Tol2* Transposons

**Figure 2:**
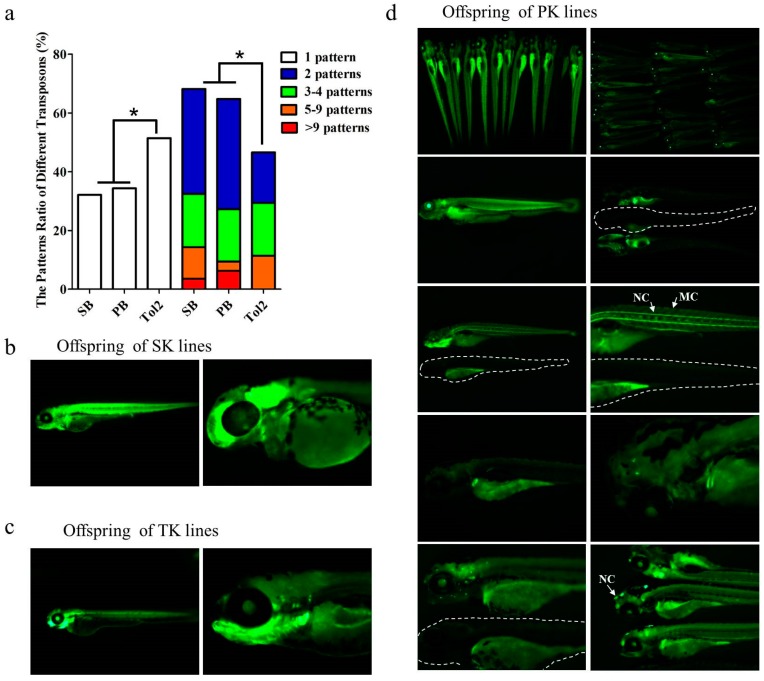
ET pattern difference based on *SB*, *PB* and *Tol2* transposons in zebrafish. (**a**) Pattern difference was measured by counting the different patterns of offspring at five days postfertilization (dpf) from each single founder. Significant difference was detected using a χ-squared test; asterisk indicates *p* < 0.05; (**b**–**d**) Offspring patterns of lines generated by SK (ET lines generated by the *SB* transposon), TK (ET lines generated by the *Tol2* transposon), and PK (ET lines generated by the *PB* transposon). In various expression patterns, specific expression in tissues such as eyes, kidney tubes, muscles, and swim bladders could be detected, as well as specific expression patterns in various cells such as nerve cells (NC) and muscle cells (MC). White dotted lines in (**d**) indicate the WT fish.

To compare the transposition frequency of three transposons in zebrafish embryos, we co-injected a mixture of ET vector and transposase mRNA of *PB*, *SB*, or *Tol2* transposon into one-cell stage embryos of zebrafish, and screened for *GFP* expression at 48 and 120 hpf. The ET vector contains an ET box with a mini-promoter Krt4, *GFP* reporter gene and β-globin polyA, and the ET box is flanked with *PB*, *SB* or *Tol2* transposon ITRs (Figure 1a). The amounts of transposase mRNA and transposon plasmid in the co-injected mixture were optimized for each transposon by measuring under fixed amount of transposon plasmids (20 ng/µL) co-injected with increasing amount of transposase mRNA (10, 30, 50, 80, 100 ng/µL). The optimal dose of *SB*, *PB*, and *Tol2* transposase was 50, 50 and 30 ng/µL (data not shown), each transposon kept the highest efficiency and survival rate under the optimal dose, and the survival rate varied between 70% and 90%, while for non-injected embryos it was around 90%. Then, we compared the differences in ET efficiency at the optimal injection dose. We found that the *Tol2* transposon represented the highest *GFP* expression (90.45%, 92.71%, N = 1524), followed by *SB* (88.64% 88.87%, N = 1378) and *PB* (83.18%, 83.18%, N = 1276) at 48 and 120 hpf, respectively, and there was a significant difference in *GFP* expression between *Tol2* and *PB* treatment (*p* < 0.05) (Figure 1b). Embryos (designated as F0) that showed *GFP* expression at 120 hpf for each transposon were raised for further germline transmission and *GFP* expression pattern analysis. The embryos surviving to adulthood were separately outcrossed with WT fish to generate F1 embryos for *GFP* screening. Various germline transfer efficiencies were observed among three transposon groups: The *Tol2* transposon represented the highest at 55.56% (N_F0_ = 165), followed by *SB* (38.36%, N_F0_ = 151), while *PB* represented the lowest at 32.65% (N_F0_ = 149), and the difference between *PB* and the *Tol2* group was significant (*p* < 0.05) (Figure 1c). The distribution of the *GFP* expression pattern of F1 embryos across the three transposon groups was also compared. We found that ET lines generated by the *Tol2* transposon (named TK) were most likely to produce offspring with a single expression pattern, while the lines generated by *PB* (named PK) and *SB* (named SK) tended to produce offspring with more expression patterns (≥2), and significant differences were observed (*p* < 0.05, X^2^ test) (Figure 2). Some representative *GFP* expression patterns of F1 embryos from *SB*, *PB* and *Tol2* transgenic lines are shown in Figure S1.

### 3.2. Annotation of Trapped Enhancers

Due to the fact that many founders (F0) of transgenic zebrafishes contained multiple insertions, which may confuse the annotation of En, *GFP*-positive F1 embryos were raised to adulthood, and outcrossed with WT fish to obtain stable transgenic F2 embryos for subsequent annotation of Ens. Seven stable F1 ET lines (four from *SB*, named SK1, SK3, SK6 and SK12; one from *PB*, named PK0, and two from *Tol2*, named TK1 and TK4) that exhibited distinct patterns of *GFP* expression were selected for further En annotation. These ET lines were separately outcrossed with WT fish to generate F2 offspring for *GFP* expression screening and insertion site annotation. The insertion sites of the seven ET lines were repeatedly determined by splinkerette PCR assay with embryos from different outcrossing (at least three times). All seven F1 ET lines were confirmed to harbor single insertion, and the insertion sites of the seven ET lines were successfully mapped to the zebrafish genome (GRCz10). These results were summarized in Table 1. In TK1, the ET cassette was inserted in exon 1 of *dlx1a* in forward direction, and two other nearby genes (*dlx2a* and *itga6a*) were also found (within 50 kb upstream and downstream of the insertion site). In TK4, the insertion was located in forward orientation in intron 1 of *rps26*, and six other endogenous genes (*arf3a*, *wnt10b*, *wnt1*, *IKZF4*, *dnajc22* and *lmbr1l*) were also located near the insertion site. In SK1, the ET cassette was inserted in intron 8 of *slc9a8* in reverse orientation, and close to the *FP102158.1* gene. In SK3, the ET cassette was located at about 20 kb downstream of *ednraa* in the forward direction. In SK6, the ET cassette was located at *mettl22* and *abat* in the reverse direction. In SK12, the ET cassette was located in intron 10 of *wdr33* in the reverse direction, and diverse endogenous genes (*si:ch1073-184j22.1*, *sft2d3*, *dusp28* and *proca*) were found nearby. The ET cassette in the PK0 line was located in intron 4 of *itgav* in the forward orientation, and in proximity to two other genes (*si:dkey-69o16.5* and *zc3h15*) (Table 1).

**Figure 3:**
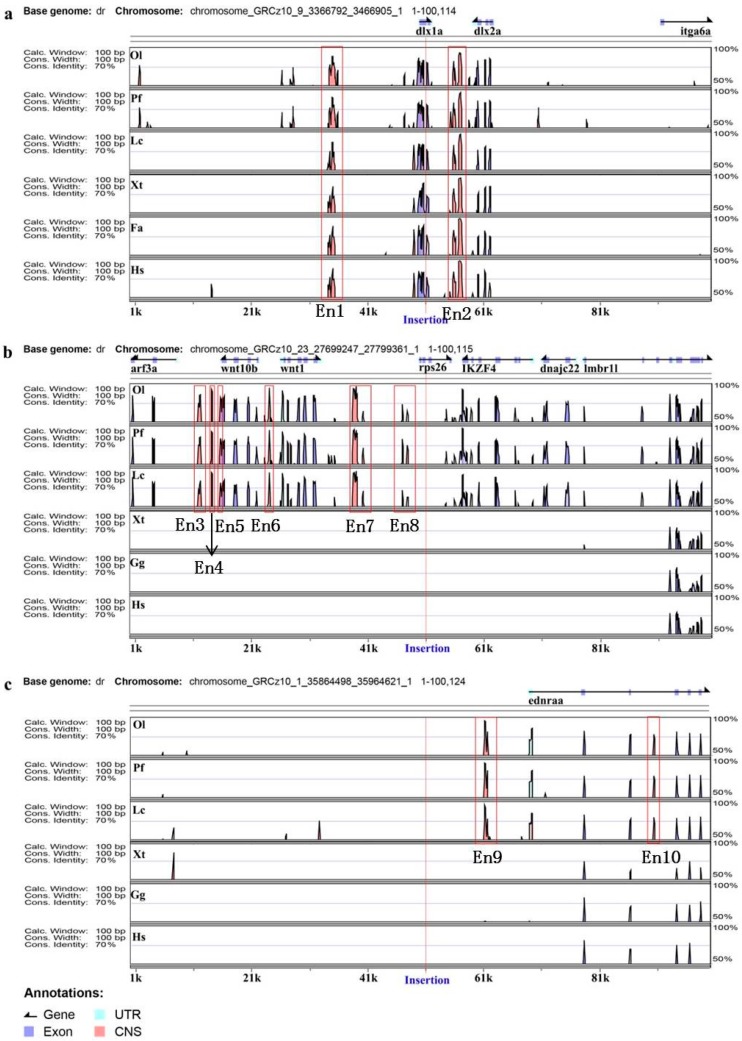
Enhancer annotation of three ET lines. Distribution in the region between 50 kb upstream and 50 kb downstream of insertion. Genomic sequences from *Danio rerio* (Dr), *Oryzias latipes* (Ol), *Poecilia formosa* (Pf), *Xenopus tropicalis* (Xt), *Ficedula albicollis* (Fa), *Gallus gallus* (Gg) and *Homo sapiens* (Hs) were analyzed in the VISTA browser (https://enhancer.lbl.gov/), and 10 enhancers (Ens) were predicted in zebrafish genome as indicated (red frame); the location of each insertion site is marked with a red line. (**a**–**c**) represent zebrafish lines TK1, TK4 and SK3, respectively.

To detect putative Ens near the integration site, an alignment analysis of genomic sequences from 50 kb upstream to 50 kb downstream of insertion site across diverse vertebrate species, including fish, amphibians, birds and mammals, was performed using VISTA (https://enhancer.lbl.gov/). A total of 10 highly conserved non-coding sequence regions, were identified within three ET lines (TK1, TK4 and SK3), based on the comparative genomic analysis, as putative Ens (Figure 3). The genome location and sequence length of these putative Ens are summarized in Table 2. Among these Ens, two (named En1 and En2) were identified in the TK1 ET line, and they were conserved across all the investigated vertebrate species (Figure 3a). En1 was located 20 kb upstream of *dlx1a*, and En2 was located between *dlx1a* and *dlx2a*. An En cluster, containing six typical Ens (En3–8) was identified in the TK4 ET line, and they were conserved across the investigated teleost species (Figure 3b). En3 was located 4 kb upstream of *wnt10b*, and En6 was located between *wnt10b* and *wnt1*, while En7 and En8 were located between *wnt1* and *rps26*, which were respectively ~11 and ~3 kb upstream of *rps26*, and ~17 and ~25 kb downstream of *wnt10b* (Figure 3b). En9 and En10 were identified in the SK3 ET line, and they were also conserved across the investigated teleost species (Figure 3c). Furthermore, En9 and En10 were located 7.5 kb upstream of *ednraa* and intron 3 of *ednraa*, respectively. Among these Ens, En1 and En2 were conserved across all the investigated vertebrate species, while the other Ens (En3–10) were conserved across all the investigated teleost species.

### 3.3. Activity Test of Identified Enhancers

**Figure 4:**
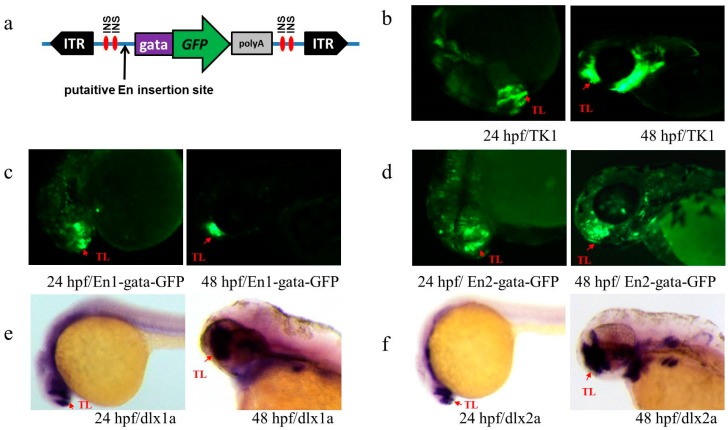
(**a**) Transposon-mediated En test vector cassette. The cassette contains an En test box flanked by two ITRs, the ITR represents *Tol2* inverted repeat sequences, and the En test box contains a minimal gata promoter, *GFP*, and β-globin polyA signal flanked with two insulators. INS represents a single insulator; (**b**–**f**) Expression patterns of Ens and endogenous *dlx1a* and *dlx2a* in TK1; (**b**) *GFP* expression of line TK1 at 24 and 48 hpf; (**c**,**d**) *GFP* expression of the embryos injected with plasmids carrying En1 and En2 at 24 and 48 hpf, respectively; (**e**,**f**) expression of *dlx1a* and *dlx2a* at 24 and 48 hpf, respectively. Red arrows indicate similar patterns in telencephalon (TL).

To determine the actual activity of these putative Ens, they were cloned and inserted into a new En test vector, which contains a trapping box flanked with two insulators (Figure 4a) intended to block the genomic position effect. Then, their En activities were evaluated in vivo by injecting the plasmids into zebrafish embryos at a one-cell stage and then screening the *GFP* signal at 24 and 48 hpf. In previous studies, we found that the Krt4 minimal promoter was still kept at a background noise even if it was flanked by double insulators. Here, we replaced the Krt4 minimal promoter by gata minimal promoter to minimize the background noise. The mRNA expression profiles of the nearest endogenous gene of these Ens were investigated by WISH.

En1, which was identified in the TK1 line, produced a distinct *GFP* expression in the telencephalon of embryos. About 45% embryos (N = 300) exhibited the specific expression at 24 hpf and 2 dpf after injection with plasmid (pEn1-gata-*GFP*) carrying the En1 (Figure 4c), which was similar to but did not match well the *GFP* expression patterns of embryos from the TK1 line (Figure 4b). The expression profile of En2, which included two conserved sequence peaks, was generally similar to that of En1 (Figure 4d), but an additional weak signal in the hindbrain was also observed. We found that when the two genomic regions corresponding to two conserved peaks of En2 were separately subcloned into the En detection plasmid, neither of them showed any En activity (data not shown). Compared with the mRNA profiles of the genes closest to En1 and En2 (*dlx1a*, *dlx2a* and *itga6a*), we found the *GFP* expression patterns of En1, En2 and TK1 lines were markedly different from *itga6a* expression, which has been described previously [37]. Expression signal of the TK1 line was not only detected in the telencephalon, but it largely mimicked that of *dlx1a* and *dlx2a* (WISH revealed that *dlx1a* and *dlx2a* were expressed in the telencephalon and other tissues including the diencephalon, prethalamus, hypothalamus, pharyngeal arch and ventral thalamus) (Figure 4e,f).

**Figure 5:**
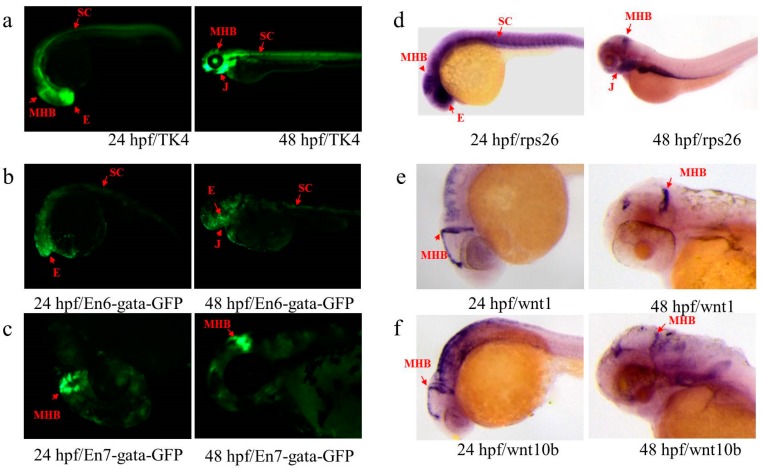
Expression patterns of En and endogenous *rps26*, *wnt1* and *wnt10b* in TK4. (**a**) *GFP* expression of line TK4 at 24 and 48 hpf; (**b**,**c**) *GFP* expression of the embryos injected with plasmids carrying En6 and En7 at 24 and 48 hpf, respectively; (**d**–**f**) gene expression of *rps26*, *wnt1* and *wnt10b* at 24 and 48 hpf, respectively. Red arrows indicate similar patterns in eye (E), midbrain and hindbrain boundary (MHB), spinal cord (SC) and jaw (J).

In the TK4 line, six putative Ens (En3–8) were identified near the insertion site, and diverse endogenous genes (*arf3a*, *wnt10b*, *wnt1*, *rps26*, *IKZF4*, *dnajc22* and *lmbr1l*) were located between 50 kb upstream and 50 kb downstream of the insertion site (Figure 3b). Two of them (En6 and En7) displayed strong En activity, while other En activity (En3–5 and En8) was extremely weak and difficult to detect (data not shown). We found that about 32% embryos (N = 280) injected with the plasmid carrying En6 (pEn6-gata-*GFP*) exhibited ubiquitous *GFP* expression at 24 hpf, and specific expression in the eyes, jaw and spinal cord at 48 hpf (Figure 5b), which was partly similar to that in the TK4 line (Figure 5a). The embryos injected with the plasmid carrying En7 (pEn7-gata-*GFP*) displayed various *GFP* expression, and about 30% embryos (N = 300) displayed strong and specific *GFP* expression in the midbrain and hindbrain boundary (Figure 5c), and were different from that in the TK4 line, but overlapped at least partially with the mRNA expression profiles of *wnt1* and *wnt10b* at 24 and 2 dpf (Figure 5d–f). We also found that none of the expression patterns of all detected endogenous genes (*rps26*, *wnt1*, *wnt10b* and *Lmbr1l*) matched well with those of the TK4 line, suggesting that this region may contain diverse regulatory elements, and each plays a role in the regulation of genes during the development of embryos, resulting in complicated expression patterns.

**Figure 6:**
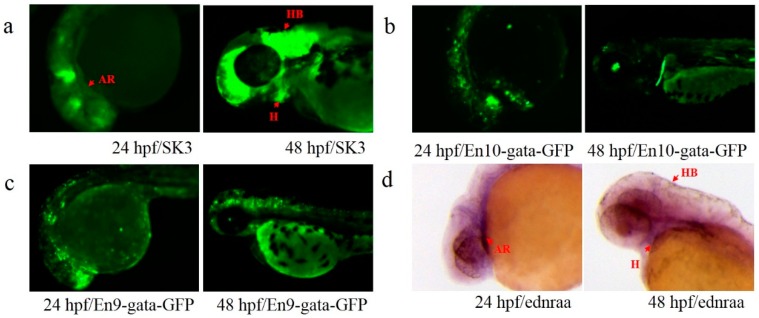
Expression patterns of En and endogenous *ednraa* in SK3. (**a**) *GFP* expression of line SK3 at 24 and 48 hpf; (**b**,**c**) *GFP* expression of the embryos injected with plasmids carrying En10 and En9 at 24 and 48 hpf; (**d**) gene expression of *ednraa* at 24 and 48 hpf. Red arrows indicate the similar patterns in arches (AR), hindbrain (HB) and heart (H).

Two putative Ens (En9 and En10) were identified in the SK3 line, and only one endogenous gene (*ednraa*) located ~15 kb downstream of the insertion site was found. En9 was located upstream (~7.2 kb) of *ednraa*, while En10 was located in intron 3 of *ednraa* (Figure 3c). Expression of *ednraa* occurred in neural crest cells of the arches at 24 hpf and in hindbrain and heart at 2 dpf (Figure 6d), which mimicked the *GFP* expression pattern of the SK3 line (Figure 6a). Both En9 and En10 displayed weak En activity, but were distinctly different from the *GFP* expression pattern of SK3 and *ednraa* (Figure 6b,c).

### 3.4. Transcription Detection of Putative Enhancers

**Figure 7:**
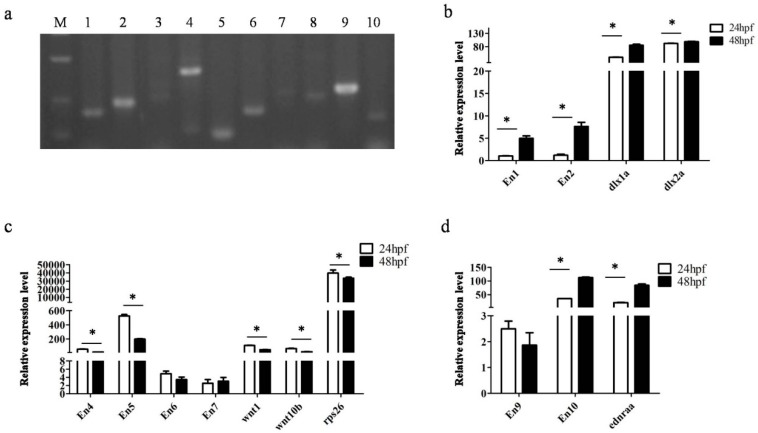
Expression of Ens. (**a**) En expression confirmed by RT-PCR, numbers 1–10 represented En1-10; M: DL500 DNA marker; (**b**–**d**) relative quantification of expressions of En and endogenous genes by qPCR at 24 and 48 hpf; error bars represent SD.

Recent studies have revealed that active Ens are often transcribed in mammalian systems, producing a class of non-coding RNAs called En RNAs (eRNAs), which have a role in the activity of Ens [38], and make the annotation of Ens more complicated. Here, we also found that the *GFP* expression patterns of some lines did not match very well with that of nearby genes, indicating these trapped Ens could express eRNAs to activate the transcription of distal genes. Then, the transcription of Ens identified in the current study was also investigated by RT-PCR and qPCR. The RT-PCR results revealed that most identified Ens (En1, En2, En4-7, En9 and En10) were transcribed in zebrafish embryos at 2 dpf, while the transcript of En3 and En8 was not detectable or very weak (Figure 7a). The qPCR results revealed that the expression dynamics of Ens were generally consistent with those of endogenous genes (Figure 7b–d). In the TK1 line, the expression of En1 and En2 was consistent with that of endogenous genes of *dlx1a* and *dlx2a*, with a higher expression at 48 hpf than at 24 hpf, and the expression levels of *dlx1a* and *dlx2a* were similar (Figure 7b). In the TK4 line, the expression of most Ens (En 4–6) and endogenous genes was similar, with an increased expression from 24 to 48 hpf (Figure 7c). In the SK3 line, the RNA expression of En9 at 24 hpf was higher than at 48 hpf, which was inconsistent with the expression of *ednraa*, while both En10 and *ednraa* displayed an increasing expression trend from 24 to 48 hpf (Figure 7d). The results also revealed that the expression levels of most intergenic Ens (En1, En2, En5, En6 and En9) were relatively lower than their nearby endogenous genes, and most En expression trends were consistent with those of their endogenous genes.

## 4. Discussion

The *Tol2, SB,* and *PB* transposon system has been widely used to create gene and En traps in diverse animal models [39,40,41,42], but the ET efficiency differences among these transposons in zebrafish has not been systematically studied [43]. Here, we took advantage of the *SB*, *PB* and *Tol2* transposon systems to introduce the ET transgene into the genome of zebrafish. Our data confirmed that in zebrafish the germline transmission of *Tol2* was the highest, and the efficiency of *PB* was the lowest. These data also demonstrated that the transposon vectors based on *SB*, *PB* or *Tol2* act as efficient En detectors in the zebrafish genome, with an ET efficiency between 32.65% and 55.56%, and the trapping efficiency of *Tol2* being the highest among the three transposons.

Here, our results also revealed that although the ET efficiency of *PB* was the lowest among the three transposons in zebrafish, it tends to produce offspring with multiple expression patterns. This suggests that *PB* inserts at multiple locations more frequently than other transposons or that the insertions are biased towards transcriptional units and TSSs, and the *GFP* reporter may be under multiple regulators, which is in agreement with the insertion preference of the *PB* transposon [32,33,34]. These data suggest that *PB* may be a reasonable option for ET in zebrafish because it tends to capture more regulatory elements compared with *SB* and *Tol2* (given that the ET efficiency at above 30% in zebrafish was still acceptable).

Ten putative Ens were identified in three transgenic lines: En1 and En2 in line TK1, En3–8 in line TK4, and En9 and En10 in line SK3. By bioinformatic analysis, we found that En3–10 were highly conserved in fish, but not in amphibians, reptiles, and mammals, while En1 and En2 were conserved in all investigated species of vertebrates. Not knowing the expression patterns of all candidate Ens in the vicinity of an insertion, we have subcloned the Ens into an En detection vector to predict the specificity of the Ens driving expression of the *GFP* reporter. An important source of noise in En detection assays is the *cis*-regulatory activity from genomic regions surrounding the transposon insertion site, also known as the position effect [44]. Here, we developed an En detection vector flanked by double insulators to reduce the position effect [45]. It is notable that the Ens may not regulate the nearest gene. For example, En6 was 26 kb upstream of *rps26*, but was closer to other genes (*wnt1* and *wnt10b*) ~2 kb distant; yet En6 displayed an expression pattern similar to that of *rps26* (Figure 5). Moreover, in line SK3, although two Ens were obtained and one of them (En10) also displayed En activity in vivo, the expression profiles did not match those of the nearest gene (*ednraa*); En10 showed a very specific expression pattern in the eye, but the closest gene, *ednraa*, was expressed in neural crest cells of the arches (Figure 6). A previous study suggested that one possible explanation could be that the mini-promoter was not compatible with the Ens of the neighbouring genes, and/or was activated by different regulatory elements [46], and that these elements may affect *ednraa* and *GFP* at long distance. It has been demonstrated that Ens can exert their effect over long distances of thousands or even hundreds of thousands of base pairs, either from upstream, downstream, or from within a transcription unit [47]. Two Ens close to *dlx1a* and *dlx2a* were obtained in zebrafish. We named them En1 and En2, which is homologous to the I12a and I12b Ens identified in mice [48,49], and the *GFP* expression pattern in transgenic TK1 embryos was similar to that of the endogenous genes (*dlx1a* and *dlx2a*), which is also generally similar to the expression pattern reported in mice [50]. The En detector vector driven by the gata mini-promoter shows that the *GFP* signal in zebrafish embryos was similar to that of *dlx1a* and *dlx2a*. TK1 was actually in *dlx1a* so it was likely that the expression of GFP in TK1 represented the combined effect of many enhancers that direct *dlx1a* expression. Therefore, it is reasonable that En1 and En2 only activated a specific GFP expression in forebrain. These data demonstrate that the En detector vector driven by the gata mini-promoter was efficient and useful for detecting putative Ens in the genome.

Based on the comparison of the expression profiles of transgenes, putatively targeted genes, and the En activity test, we found that four Ens showed similar activity to expression of nearby genes. Activity of other transgenes did not match the expression of nearest genes, and some cloned Ens (En3–5 and En8) did not even show any *GFP* expression signal in the En activity test, indicating the interaction between En and targeting gene is more complicated. Other genes in the surrounding can also be targets as suggested by Harmston [51], and the Ens may act as distal regulatory elements. It has been suggested that Ens work within the context of chromatin domains, and they may regulate genes located one megabase or more from them [52] (e.g., 20% to 30% of *Drosophila* Ens may act as distal elements [53,54,55]). A variety of contrasting mechanisms of En function have been proposed over the years, including En tracking, linking and looping. Thus, defining the trapped Ens and identification of their target genes are still very challenging, especially if Ens act as distal elements or are in potentially larger regulatory blocks. The efficiency of the current ET method, and annotation of Ens is very limited and needs to improve significantly. For ET vectors, it may help to block the upstream or downstream position effect by introducing insulators.

In addition, the activity of some Ens, not matching the expression of nearby genes, may be related to functioning of these Ens acting through eRNA. The expression of eRNAs have been independently confirmed in many different cell types and species, suggesting that eRNA synthesis is not unique to neurons as previously thought, but more likely represent a universal cellular mechanism involved in governing En function [56]. Recent studies have provided more direct evidence that at least some eRNAs are functionally important for target gene expression. The knockdown of several eRNAs caused decreased expression of target genes [57,58,59]. In the present study, we found that most Ens can be transcribed, suggesting that the transcription of En may be a common phenomenon [38]. The functional properties of these eRNAs and their interaction with target genes warrant further exploration.

## 5. Conclusions

We conducted a comparative study of the ET efficiency using *PB*, *SB*, and *Tol2* transposons, and demonstrated that the ET system mediated by these transposons is highly efficient, with *Tol2* having the highest efficiency. We identified 10 putative Ens based on the seven transgenic ET lines mediated by *SB*, *PB*, and *Tol2*, and four of them displayed robust enhancer activity upon placement in the En test vector. Comparison of the *GFP* expression pattern and WISH results suggest that En1 and En2 may be involved in the regulation of *dlx1a* and *dlx2a* expression. En6 may be involved in regulation of the expression of line TK4 transgene and *rps26*, and En7 may be involved in regulation of the expression of *wnt1* and *wnt10b*. En1, En2, En6, En7 and En10 are transcribed at an early stage of zebrafish embryo development, and may regulate target genes by eRNA-based mechanism.

## Figures and Tables

**Table 1:** Insertion sites in seven ET lines.

ET Lines	Nearest Gene	Other Genes within 50 kb Upstream and Downstream Regions	Chromosome	Insertion Position Relative to Gene	Orientation in Genome
TK1	ENSDARG00000013125 (*dlx1a*)	*dlx2a*, *itga6a*	9	Exon 1	Forward
TK4	ENSDARG00000037071(*rps26*)	*arf3a*, *wnt10b*, *wnt1*, *IKZF4*, *dnajc22*, *lmbr1l*	23	Intron 1	Forward
SK1	ENSDARG00000020699 (*slc9a8*)	FP102158.1	23	Intron 8	Reverse
SK3	ENSDARG00000011876 (*ednraa*)	–	1	20 kb (upstream)	Forward
SK6	ENSDARG00000077863 (*mettl22*)	*abat*, CU464120.3	3	Exon 12	Reverse
SK12	ENSDARG00000018272 (*wdr33*)	si:ch1073-184j22.1, *sft2d3*, *dusp28*, *proca*	2	Intron 10	Reverse
PK0	ENSDARG00000006314 (*itgav*)	si:dkey-69o16.5, zc3h15	9	Intron 4	Forward

**Table 2:** The detailed information of 10 Ens.

Name	ET Lines	Location	En Length (bp)	En Position Relative to the Insertion
En1	TK1	chromosome_GRCz10_9_3366792_3466905_1:34392-35394	1003	Upstream
En2	TK1	chromosome_GRCz10_9_3366792_3466905_1:55781-57232	1452	Downstream
En3	TK4	chromosome_GRCz10_23_27699247_27799361_1:11781-12216	436	Upstream
En4	TK4	chromosome_GRCz10_23_27699247_27799361_1:14057-14217	161	Upstream
En5	TK4	chromosome_GRCz10_23_27699247_27799361_1:15551-15703	153	Upstream
En6	TK4	chromosome_GRCz10_23_27699247_27799361_1:23944-24162	219	Upstream
En7	TK4	chromosome_GRCz10_23_27699247_27799361_1:38416-39198	783	Upstream
En8	TK4	chromosome_GRCz10_23_27699247_27799361_1:46874-47866	993	Upstream
En9	SK3	chromosome_GRCz10_1_35864498_35964621_1:61133-61728	596	Downstream
En10	SK3	chromosome_GRCz10_1_35864498_35964621_1:90391-90590	200	Downstream

## Supplementary Materials

The following are available online at http://www.mdpi.com/2073-4425/9/12/630/s1, Figure S1: Representative *GFP* expression patterns of F1 embryos. (a–e) *GFP* expression of lines TK1, TK4, SK1, SK3 and PK0 at various developmental stages (1 dpf, 2 dpf, 3 dpf, 4 dpf and 5 dpf), respectively. Table S1. Primers used for the splinkerette PCR assay and En cloning, Table S2: Primers used for qPCR to investigate the transcription of the identified Ens and endogenous genes.
