# Supplementary material for: Enhancer Trapping and Annotation in Zebrafish Mediated with Sleeping Beauty, piggyBac and Tol2 Transposons
Source: Genes (Basel). 2018 Dec 13;9(12):630. doi: 10.3390/genes9120630 (PMC6316676; doi:10.3390/genes9120630)
Supplement: Supplementary file 1 [file genes-09-00630-s001.zip › Additional file 2. Supplementary Table 1.pdf]

Primers	From 5′ to 3′
SPLINK1	CGAAGAGTAACCGTTGCTAGGAGAGACC
SPLINK2	GTGGCTGAATGAGACTGGTGTCGAC
SB-1R	TCCAAGCTGTTTAAAGGCACAGTCA
SB-2R	TCATGCACAAAGTAGATGTCC
PB-1R	ACTTTATAGAAGAAATTTTGAGTTTTTGTT
PB-2R	GTTGAATTTATTATTAGTATGTAAGTGT
Tol2-1R	TCAAAGTCCCTCTGCGTGTC
Tol2-2R	GCTGGCTGACCAATTTTATACACACTC
T1	TGCTCTCACACACACACAAG
En1F	GG*GAATTC*GGAATAAAAGAAAAAGCAAAGC
En1R	CC*ACCGGT*TCTCTCCATCCCCTTTAG
En2aF	GG*GAATTC*AGAGGGTCAGCGTTATTTCACTCT
En2aR	CC*ACCGGT*GCCTTCATCACTCCGACATTGT
En2b3F	GG*GAATTC*CAGGAATGAAAAATCCTGCTTTATTTTCCC
En2b3R	CC*ACCGGT*TGTAGACAACTTGAAAAATGCCATACCC
En3F	GG*GAATTC*TTTGATCGATGTGTTTATCA
En3R	CC*ACCGGT*GCTCCTTGTCCAGCTTCCC
En4F	GG*GAATTC*TGGTCTACGACCATCCAATT
En4R	CC*ACCGGT*AGAGGACTTGAGAAAGAGT
En5F	GG*GAATTC*AGGCTGAAGATTCCAAGCGG
En5R	CC*ACCGGT*TATCCGTTTCTCTCCTACTT
En6F	GG*GAATTC*AAGCTCTGGCAACGAAAAGT
En6R	CC*ACCGGT*TATTTAATGAGGGCTGCTT
En7F	GG*GAATTC*AGGGAGGCAGAGGGTGGA
En7R	CC*ACCGGT*CCCTCTCCCTGCTTGCCG
En8F	GG*GAATTC*TAATTATTGGCTATGTTTTTACAGCAA
En8R	CC*ACCGGT*GTACTTATGCAAGCTGCTCTC
En9F	GG*ACCGGT*ATCTCTCTCTCTCTTTTGTTTTTTG

En9R	CC*ACCGGT*AATTGTGACATAATTGGGTAATACTAA
En10F	GG*GAATTC*CTTTCTCATCCGGCAAAGT
En10R	CC*ACCGGT*TTCCTTTGCTCATAGTCAT
