# Supplementary material for: Enhancer Trapping and Annotation in Zebrafish Mediated with Sleeping Beauty, piggyBac and Tol2 Transposons
Source: Genes (Basel). 2018 Dec 13;9(12):630. doi: 10.3390/genes9120630 (PMC6316676; doi:10.3390/genes9120630)
Supplement: Supplementary file 1 [file genes-09-00630-s001.zip › Additional file 3. Supplementary Table 2.pdf]

Primers	From 5′ to 3′
qEn1F	CACAAAAGCACCGTCCATGG
qEn1R	GGTCAGTACTCCAGCACACC
qEn2F	TCTCCTCTTGATTACGCGCC
qEn2R	TATAACTACCGCTGCGCACC
qEn3F	AGAGAGAGGGAGTGGAGCAG
qEn3R	CAAGCCTAACCAACCCGACT
qEn4F	GGGAGAAACAGCGACAGGAA
qEn4R	TGAGAAAGAGTGGCGTGGAA
qEn5F	AGGCTGAAGATTCCAAGCGG
qEn5R	GCGTCGTGTTGCTGTTGTTA
qEn6F	CATTTTGCCTTGGCGTGTAT
qEn6R	ATGAGGGCTGCTTTTCTAGG
qEn7F	TGCAAGTAGGTGATCGGCAG
qEn7R	CAACCCACACACTCTCTCCC
qEn8F	CAACTGACCCAATCGAGGCT
qEn8R	GACTGTCCCCTGTGTCACTG
qEn9F	GCGTTCATCCCACTCAGTCA
qEn9R	AGCGGGAGTTGAGTTTGGAG
qEn10F	CTTTCTCATCCGGCAAAGTGG
qEn10R	CTCTCGTGGCTGTTTCGGTA
EF1 F	GATGCACCACGAGTCTCTGA
EF1 R	TGATGACCTGAGCGTTGAAG
dlx1a F	CCCAGACCAGATGTGATCCG
dlx1a R	AAGAGTCACGGAAAGGGCAG
dlx2a F	ACCGATGCCACGTTGATCAT
dlx2a R	GTGCTCTGATTCACCCTGCT
rps26 F	AACGGTCGTGCTAAGAAGGG
rps26 R	GTGGTGTACGGTCCTTCCTG
ednraa F	TGACTGACTGACGGGATCCT
ednraa R	CATCCTGGGCTTCCTCTGTG
wnt1 F	ACCAGAAAACCCAGCGCATA
wnt1 R	CTTTCGGTGACCTGCTCCAT
wnt10 F	TCACGGTACTGGGCAATGAC
wnt10 R	CCCCTGTTGAGAATGGCACT
