# Supplementary figures and images for: Enhancer Trapping and Annotation in Zebrafish Mediated with Sleeping Beauty, piggyBac and Tol2 Transposons
Source: Genes (Basel). 2018 Dec 13;9(12):630. doi: 10.3390/genes9120630 (PMC6316676; doi:10.3390/genes9120630)

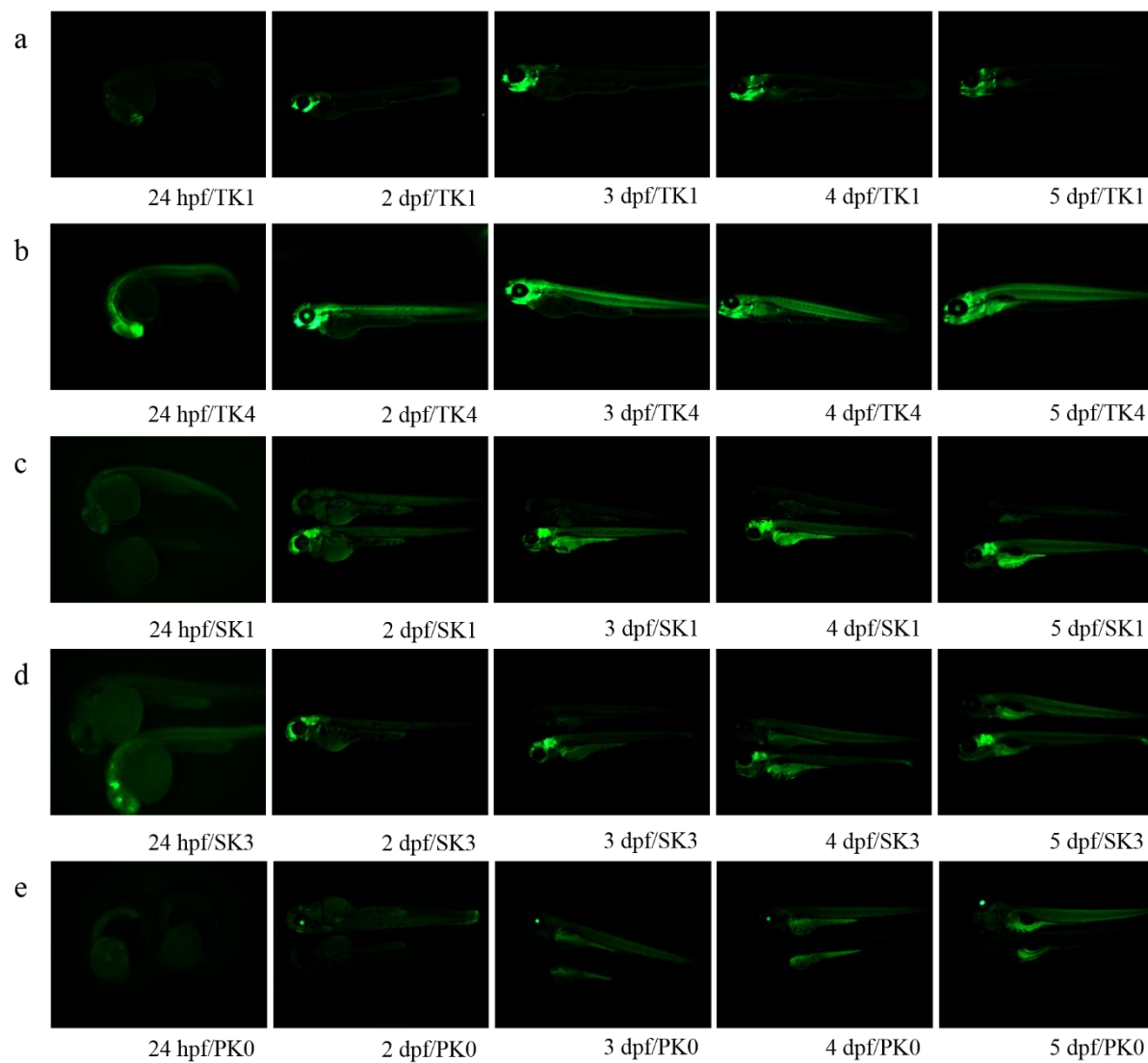

Supplement: Supplementary file 1 [file genes-09-00630-s001.zip › Additional file 1. Supplementary Figure S1.pdf]
